# Identification of three novel QTL for resistance to highly aggressive Canadian strains of *Plasmodiophora brassicae* in rutabaga cultivar ECD10

**DOI:** 10.3389/fpls.2025.1588460

**Published:** 2025-06-30

**Authors:** Md. Masud Karim, Fengqun Yu

**Affiliations:** Saskatoon Research and Development Centre, Agriculture and Agri-Food Canada, Saskatoon, SK, Canada

**Keywords:** *Brassica napus*, *Plasmodiophora brassicae*, clubroot resistance, genotyping-by-sequencing (GBS), GBS-SNP-CROP, genetic mapping, QTL, candidate gene

## Abstract

*Plasmodiophora brassicae*, the causal pathogen of clubroot, has emerged as a significant threat to the production of Canada’s most important oilseed crop canola. The *B. napus* rutabaga cultivar ‘Wilhelmsburger’ (ECD10) has shown a high resistance level to Canadian *P. brassicae* strains. This study utilized genotyping-by-sequencing (GBS) to identify single-nucleotide polymorphism (SNP) markers for mapping QTL related to resistance to 11 P*. brassicae* strains in a BC_1_/BC_1_S_1_ population. The short reads from the GBS were assembled to the Chinese canola cultivar ‘ZS11’ reference genome. A genetic map spanning 1,812.6 cM was developed using 1,049 non-redundant SNPs identified from 92 BC_1_ plants. Three quantitative trait loci (QTL) were identified: *Rcr13* on chromosome A02, *Rcr11* on A08, and *Rcr_C03-4^ECD10^* on C03. *Rcr13* was related to resistance to four strains (2B, 8J, 5L, and 5X), explaining 14.2% to 81.7% of the phenotypic variation explained (PVE). *Rcr11* was associated with resistance to eight strains (3A, 2B, 5C, 3D, 8E, 5G, 3O, and 8P), explaining 20.2% to 83.2% of the PVE. *Rcr_C03-4^ECD10^* was linked to resistance against strain 5C, explaining 21.5% of the PVE. Thirteen genes that encode proteins related to disease resistance were found as candidate genes for three QTL. The syntenic regions of the QTL were also examined with the reference genomes of the *B. rapa* `Chiifu', *B. oleracea* `D134' and eight different *Brassica napus* 'Darmor', `Gangan', `No2127', `Quinta', `Shengli', `Tapidor', `Westar', and `Zheyou7'. Differential reactions of the *B. napus* line carrying *Rcr11* or *Rcr13* with those carrying the previously identified CR genes *Rcr3* and *Rcr9* or *Rcr8* were found with the purified *P. brassicae* isolates. This is the first report of rutabaga-derived QTL effective against Canadian pathotypes that overcome ‘Mendel’ resistance.

## Introduction

*Brassica napus* (canola or oilseed rape) is the most extensively cultivated oilseed crop in Canada, producing around 20 million tons annually and contributing $43.7 billion to the Canadian economy each year (Canola Council of Canada, 2025). Clubroot caused by *Plasmodiophora brassicae* Woronin, a soil-borne and biotrophic protist, is a major threat to canola production. Thousands of infested canola fields have been identified across the country (Froese et al., 2019) since it was first discovered in a commercial canola field in Alberta in 2003 (Tewari et al., 2005).

The disease impacts the production of brassica crops in over 60 countries (Dixon, 2009). When club- or spindle-shaped galls form on roots, the pathogen hampers nutrient and water uptake, impairing proper plant growth and leading to significant yield loss. The use of clubroot-resistant (CR) cultivars is the most effective and economical way to control this disease (Karim et al., 2020). However, the repeated use of the same CR source for breeding is not a sustainable approach, as it enables minor pathotypes to become predominant (Cao et al., 2020; Sedaghatkish et al., 2019). In Canada, breeding companies launched CR canola cultivars in 2009 and 2010. These cultivars became susceptible in 2013 with the emergence of the new virulent pathotype 5X (Strelkov et al., 2016). Most CR cultivars in Canada carry a resistance gene from the oilseed rape cultivar ‘Mendel’ (Fredua-Agyeman et al., 2018). However, strains aggressive to the first generation of CR cultivars have been identified from the canola fields in Canada. The *P. brassicae* strains collected in the Canadian prairie have been classified into more than 30 pathotypes using the Canadian Clubroot Differential (CCD) set (Strelkov et al., 2018; Hollman et al., 2021). To control clubroot disease effectively, gene pyramiding from diverse resources should be considered.

Researchers have identified numerous quantitative trait loci (QTL)/genes associated with clubroot resistance in *B. napus*. Key findings include a major resistance gene against race ‘C’ or ‘N4’ from *B. napus* subsp*. napobrassica* cv. ‘Wilhelmsburger’, and another major gene for race ‘B’ or ‘N4’ from *B. napus* subsp. *pabularia* cv. ‘NZ’ (Johnston, 1970; Lammerink, 1967). Additionally, two major resistance genes for races 2 and 3 of *P. brassicae* were found in *B. napus* subsp. *napobrassica* cv. ‘York’ (Ayers and Lelacheur, 1972). The ‘Wilhelmsburger’ cultivar was also found to contain a major gene for race 3 and two major genes for race 2 (Ayers and Lelacheur, 1972). In *B. napus* subsp. *oleifera* cv. ‘Darmor-bzh’, two QTL for K92–16 and a major gene *Pb-Bn1* to Pb137-522, were identified (Manzanares-Dauleux et al., 2000). Resynthesized *B. napus* revealed 19 QTL for resistance to various isolates, located on chromosomes A02, A03, A08, A09, C05, C06, and C09 (Werner et al., 2008). A single major gene on A08 conferred resistance to several pathotypes in *B. napus* subsp. *napobrassica* cv. ‘Brookfield’ (Hasan and Rahman, 2016). The *CRa* gene, associated with resistance to pathotype 3, was identified on A03 in *B. napus* subsp. *oleifera* line 12-3 (Zhang et al., 2016). Another dominant resistance gene for pathotype 3 was found on A03 in *B. napus* subsp. *oleifera* cv. ‘Mendel’ (Fredua-Agyeman et al., 2016). Two QTL for pathotype ECD 17/31/31 were mapped on A02 and A03 with a genetic diversity panel comprising 245 *B. napus* accessions (Hejna et al., 2019). The A03 genomic region was identified for resistance to several pathotypes (Fredua-Agyeman et al., 2021). A genome-wide association study involving 177 *B. napus* accessions was employed to identify QTL for resistance to clubroot pathogen along with corresponding SNPs: 2 SNPs for 5X, 4 SNPs for 3A, 5 SNPs for 2B, and 6 SNPs for 3D (Dakouri et al., 2021). In *B. napus* lines with clubroot resistance introgressed from the turnip cultivar ECD01, two major QTL, *Rcr10^ECD01^* on A03 and *Rcr9^ECD01^* on A08, were identified, conferring resistance to strains of pathotypes 3A, 3D, 3H, 5X (Yu et al., 2021). Lastly, a dominant locus for resistance to pathotypes 2, 4, 7, and 11, as well as PbGh (Pb3), was mapped to chromosome A03 in the *B. napus* mutation breeding line ‘Kc84R’ (Jiang et al., 2022).

CR sources in canola (*B. napus*, AACC) are extremely limited. Great efforts have been put into searching for resistance in the diploid progenitors *B. rapa* (AA) and *B. oleracea* (CC), but the introgression of CR genes from these diploid species into *B. napus* is not straightforward. Recently, resynthesized *B. napus* with race-specific resistance genes and race-non-specific QTL to multiple races of *Plasmodiophora brassicae* have been developed (Karim and Yu, 2024). The CR oilseed rape cultivar ‘Mendel’ originating from ECD04 (*B. rapa* subsp*. rapifera*) and ECD15 (*B. oleracea* subsp. *capitata*) (Diederichsen and Sacristan, 1996), has been widely used as the primary source of resistance for developing CR canola cultivars in Canada. Genetic mapping showed that the hybrid ‘Mendel’ has one dominant CR gene on A03 (Fredua-Agyeman and Rahman, 2016) and another gene on A08 for resistance to Canadian strains of *P. brassicae* (Rahaman, 2022). ‘Mendel’ showed good resistance against field isolates from Germany, while the *B. napus* differential line ECD10 (*B. napus* subsp. *napobrassica* ‘Wilhelmsburger’) was reported to be susceptible (Diederichsen and Sacristan, 1996). Interestingly, ECD10 showed resistance against all 17 pathotypes in the CCD set, while ‘Mendel’ showed resistance to 13 pathotypes (Strelkov et al., 2018). Therefore, the genetic basis of resistance in ECD10 is likely distinct from that in ‘Mendel’.

As mentioned earlier, the inheritance of resistance to clubroot in ECD10 has been studied by a few researchers using different pathotypes of *P. brassicae* (Lammerink, 1967; Johnston, 1970; Ayers and Lelacheur, 1972). However, genetic mapping of resistance genes in ECD10 has not been performed previously. Since ECD10 demonstrated strong resistance to Canadian pathotypes of *P. brassicae* (Strelkov et al., 2018), it is crucial to identify CR genes, conduct genetic mapping of resistance genes, and develop DNA markers linked to each gene in use of marker-assisted breeding. In this study, a BC_1_ mapping population was developed by crossing the resistant rutabaga cultivar ECD10 with the susceptible canola breeding line DH16516. Genotyping-by-sequencing (GBS) was performed on the BC_1_ plants, and BC_1_S_1_ families were obtained through self-pollination of BC_1_ plants for phenotyping. In this study, we aimed: 1) to examine DNA variants genome-wide in the BC_1_ progenies using GBS; 2) to identify QTL linked to resistance against 11 strains representing 11 pathotypes of *P. brassicae*; 3) to identify potential candidate resistance genes related to the QTL; 4) to address the relationship of the CR genes in ECD10 with the previously identified CR genes.

## Materials and methods

### Development of a segregating population

Seeds of ECD10, a rutabaga (*B. napus*) cultivar carrying CR genes, were kindly provided by Dr. G. R. Dixon (The University of Warwick, Wellesbourne, Warwick, UK). DH16516 (spring-type, clubroot-susceptible, canola-quality *B. napus*) line was developed by Dr. Séguin-Swartz at the Saskatoon Research and Development Centre (SRDC), Agriculture and Agri-Food Canada (AAFC), Saskatoon, Saskatchewan, Canada. DH16516 was pollinated with ECD10 to generate F_1_ progeny and backcrossed with DH16516 to produce BC_1_ plants. Ninety-two BC_1_ plants were selected to produce 92 BC_1_S_1_ families through self-pollination in a greenhouse at the SRDC.

Our group has developed spring-type *B. napus* breeding lines DH10-45, DHY427, DHY644, DHM1510, and DHY154M21 carrying single clubroot resistance genes *Rcr13* and *Rcr11* (identified from this study), *Rcr8* (Yu et al., 2017), *Rcr3* (Rahaman, 2022) and *Rcr9* (Yu et al., 2021) respectively. The *B. napus* lines were used for evaluating their differential reactions against the purified *Plasmodiophora brassicae* isolates.

### Evaluation of plants for resistance to clubroot

Dr. S. Strelkov (University of Alberta, Canada) kindly provided 11 strains representing 11 pathotypes characterized by the CCD set in his group (Strelkov et al., 2018). Clubroot galls were produced by inoculating and maintaining seedlings of the canola breeding line Y549-(0)-2-1, which contains the *Rcr1* gene (Hu et al., 2024). The inoculum preparation, inoculation method, and growth condition were the same as those outlined by Yu et al., 2021. The parents (DH16516 and ECD10) and 92 BC_1_S_1_ families were assessed with 11 strains F.3-14 (pathotype 3A), F.183-14 (2B), F.175-14 (5C), F.1-14 (3D), F.187-14 (8E), CDCS (5G), F.12-15 (8J), CDCN#2 (5L), F.381-16 (3O), Leduc C#37 (8P), and LG-02 (5X). Twelve seeds from each family were evenly planted in two pots, which were then inoculated with 15 ml of inoculum (1 × 10^7^ spores/ml) per pot at seven days after sowing. The plants’ leaves were pruned regularly starting two weeks after sowing. Watering was stopped two days prior to the clubroot rating. Disease reaction was rated at six weeks after inoculation using the 0–3 scale described by Kuginuki et al. (1999). The DSI for each family was calculated using the method described by Horiuchi and Hori (1980).

Each family showing a resistance response (DSI ≤ 60%) in the initial study was reassessed two more times. For families with inconsistent results, the highest DSI from the three assessments was considered the most reliable and used to determine the families’ resistance response. BC_1_S_1_ families with a DSI ≤ 60% were classified as R, while those with a DSI > 60% were classified as S (Yu et al., 2017).

Our group has developed a protocol for purifying the genotype of *P. brassicae* strains (unpublished). Twelve plants in each of the spring-type breeding lines were assessed with the purified isolates Pb-SPI-33, Pb-SPI-87, Pb-SPI-110, and Pb-SPI-151 respectively to identify differential reactions among the lines. The experiments were repeated twice.

### Genotyping-by-sequencing of 92 BC_1_ plants and parental lines

Young leaves from the 92 BC_1_ plants and two replications of the two parents were collected and dried for 48 hours in a Freezone 6 dryer (Labconco Corp, Kansas City, MO). DNA extraction was as described previously (Karim and Yu, 2023). In total, 96 DNA samples including two replications of the parents DH16516 and ECD10 were prepared for GBS. The preparation of GBS libraries was conducted at the Institute of Integrative Biology and Systems (IBIS) (Pavillon Charles-Eugène-Marchand, 1030 Avenue de la Médecine, Université Laval, Québec, G1V 0A6, Canada). Quality control (QC) of the Illumina library and sequencing on the Illumina HiSeq2500 PE125 platform were performed at Genome Quebec (630 Boul Rene-Lévesque O, Bureau 2660, Montreal QC H3B 1S6, Canada).

### Alignment of GBS short reads and SNP calling

Short reads from each of the 92 BC_1_ samples, the parents ECD10 and DH16516 were assembled to the *B. napus* ‘ZS11’genome (Song et al., 2020). The genome sequence was obtained from the *B. napus* Pangenome Information Resource (BnPIR) (hzau.edu.cn). The NGS pipeline GBS-SNP-CROP v3.0 (Melo et al., 2016) was used for alignment and variant identification on the AAFC Biocluster Linux platform. The process for the alignment of DNA short reads and SNP calling followed the method described previously (Karim and Yu, 2023). An SNP panel from GBS-SNP-CROP of 92 BC_1_ plants along with the parents ECD10 and DH16516 was used to identify polymorphic SNP sites using TASSEL5 (Bradbury et al., 2007). KNNimp imputation was performed with default parameters: high LD sites = 30, number of nearest neighbors = 10, and maximum distance between sites for finding LDs = 10,000,000 to recover missing data. The data were filtered with a minimum count of 5, a minimum frequency of 0.1 (10%), a maximum heterozygosity frequency of 1.0, and a maximum frequency of 1.0 (100%). SNP panels from two replicates each of DH16516 and ECD10 were combined, and sites common to both lines were retained. Then, the common SNP panels of ECD10 and DH16516 were used to identify polymorphic SNP sites in 92 BC_1_ plants. SNP alleles from DH16516 (S) were assigned the score ‘A’, those from ECD10 (R) were scored as ‘B’, and the heterozygous allele was scored as ‘H’. Since DH16516 is a homozygous line, all SNP sites in the BC_1_ population should theoretically show either the recipient parent allele (A) or the heterozygous allele (H). The ‘B’ allele and SNPs with more missing data were excluded using the Excel sort function.

### Construction of linkage map and identification of QTL

A two-step filtering process was conducted using JoinMap 4.1 (Van Ooijen, 2011) and the BIN function of ICIMapping (Meng et al., 2015) for eliminating redundant markers. The filtered SNP sites from JoinMap 4.1 were then used for binning redundant markers, constructing a genetic map, and mapping QTL associated with resistance to clubroot using Inclusive Composite Interval Mapping (ICIM) method of the QTL IciMapping software (Meng et al., 2015). MapChart 2.1 (Voorrips, 2002) was employed to visualize the linkage map. The LOD score threshold was determined through a 1,000-permutation test with a Type I error rate of 0.05 to identify QTL. The effects of the QTL were assessed in terms of phenotypic variation explained (PVE) and additive effects (Add) values for each QTL.

### Identification of potential candidate genes in the intervals of QTL

The coding sequences (CDSs) of genes located within the discovered QTL intervals were retrieved from the *B. napus* ‘ZS11’ genome for gene annotation using Blast2GO (Conesa et al., 2005). Genes encoding disease-resistance proteins were identified based on Blast2GO descriptions. These CDSs were further analyzed using BLAST at www.arabidopsis.org to find homologous genes in Arabidopsis and classify the types of corresponding disease-resistance proteins. Homology was determined based on the highest nucleotide sequence similarity and the longest alignment length.

### Search for syntenic regions of disease resistance genes in ‘Chiifu’ and ‘D134’ genomes

The *B. rapa* reference genome ‘Chiifu’ version 3.0 (Zhang et al., 2018) was obtained from https://brassicadb.org/brad/downloadOverview.php and used to assess the synteny of disease resistance-related genes found in *B. napus* ‘ZS11’ through the BLAST search tool at http://brassicadb.cn/#/BLAST/. Additionally, the *B. oleracea* reference genome ‘D134’ (Lv et al., 2020) was obtained from https://db.cngb.org/search/?q=CNP0000469 and analyzed for synteny of the disease resistance-related genes identified in *B. napus* ‘ZS11’ using MegAlign Pro 15 with the MAUVE tool (DNASTAR, Madison, WI, United States).

### Resistance gene analogue across different *Brassica napus* reference genomes

Eight different *Brassica napus* reference genomes—’Darmor-bzh-v10’, ‘Gangan’, ‘No2127’, ‘Quinta’, ‘Shengli’, ‘Tapidor’, ‘Westar’, and ‘Zheyou7’—were downloaded from https://bnaomics.ocri-genomics.net/ and analyzed using the RGAugury pipeline (Li et al., 2016) for genome-wide prediction of RGAs.

## Results

### Inheritance of clubroot resistance in the rutabaga cultivar ECD10

**Figure 1:**
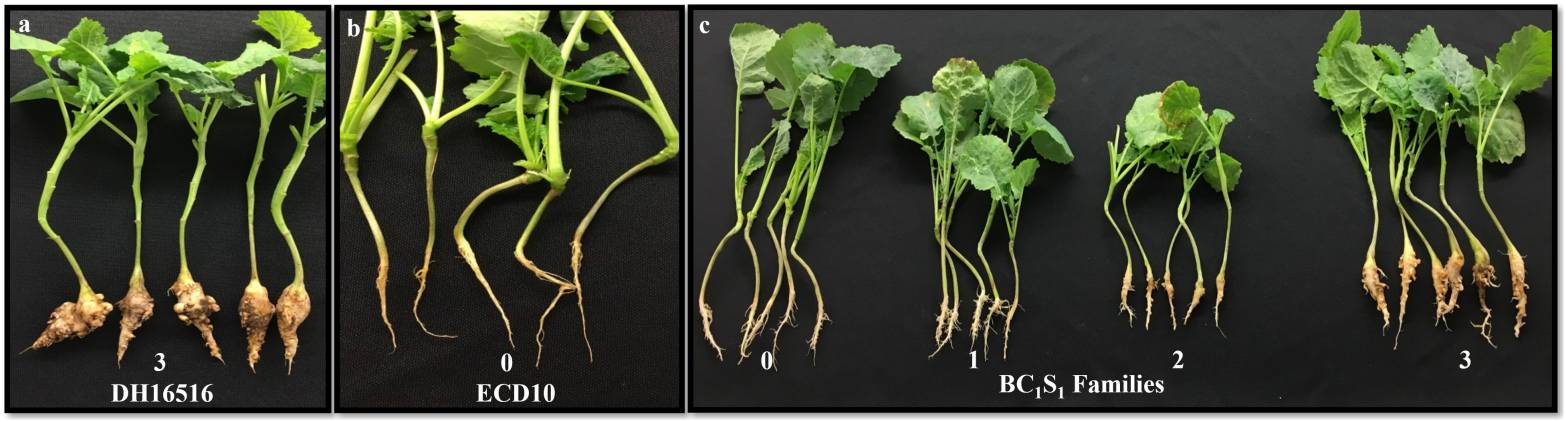
Test of BC_1_S_1_ families from BC_1_ plants [DH16516 × (DH16516 × ECD10)] against 11 pathotypes of *Plasmodiophora brassicae*; **(a)** phenotypes of the susceptible parent DH16516, **(b)** resistant parent ECD10 and **(c)** BC_1_S_1_ families.

**Figure 2:**
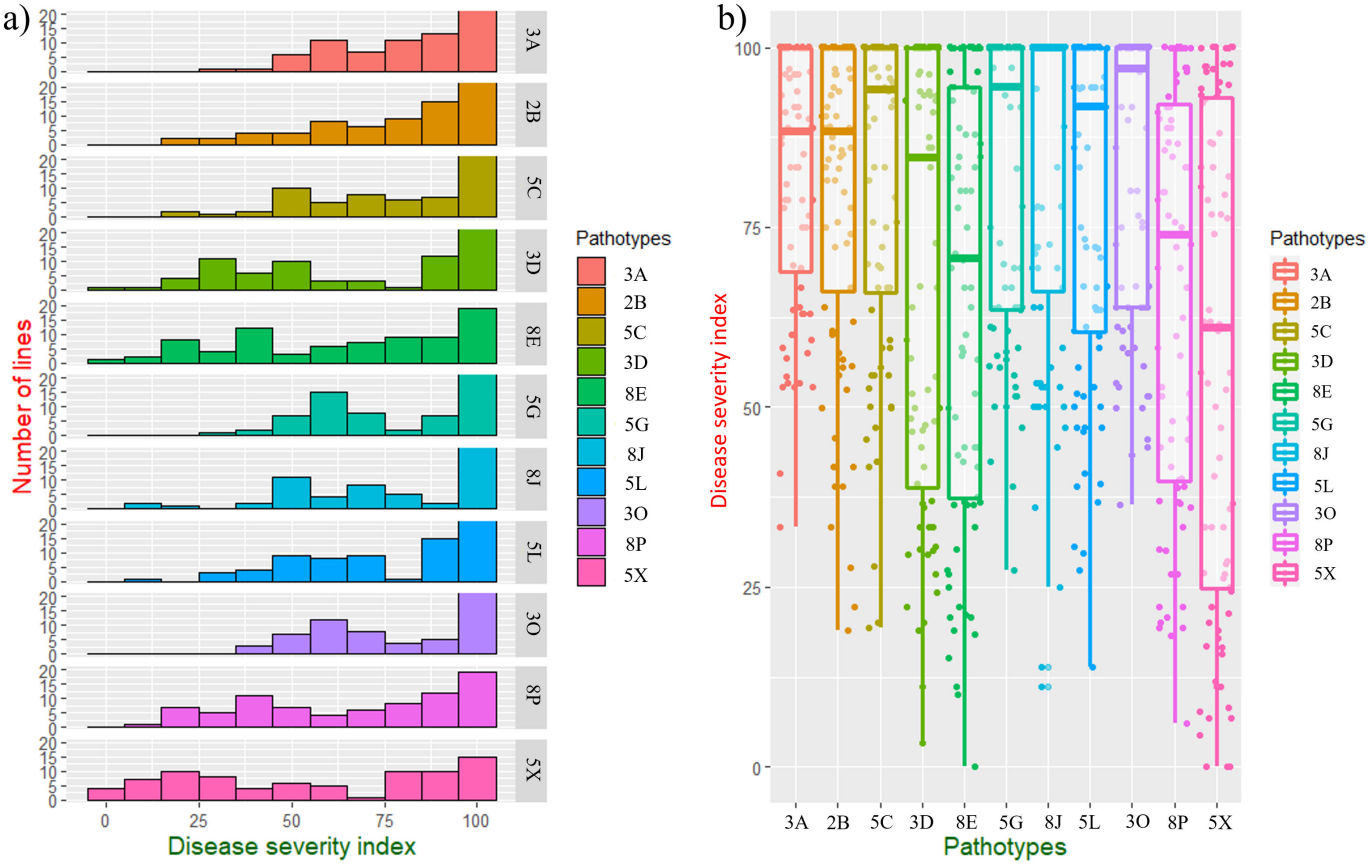
Evaluation of BC_1_S_1_ families derived from [DH16516 × (DH16516 × ECD10)] against 11 pathotypes of *Plasmodiophora brassicae*; **(a)** distribution of disease severity indexes (DSIs) of 92 families against 11 pathotypes, **(b)** boxplots showing phenotypic variation to 11 pathotypes in 92 BC_1_S_1_ families.

Resistance to clubroot in the parents (ECD10 and DH16516) as well as 92 BC_1_S_1_ families was evaluated against 11 strains classified into 11 pathotypes (Strelkov et al., 2018) (**Figure 1**; **Table 1**). ECD10 exhibited a high level of resistance to all of the strains or pathotypes with a disease severity index (DSI) of 0–19.4%, while DH16516 was highly susceptible to them (94.4–100.0% DSI). The distribution of clubroot DSI across each strain in the BC_1_S_1_ families was classified into two groups: resistant (R) families with DSI ≤ 60% and susceptible (S) families with DSI > 60%. Boxplots showing the phenotypic variation to 11 strains in 92 BC_1_S_1_ families are presented in **Figure 2**. The segregation for R and S ratio was 1:1 for 4 strains (3D, 8E, 8P, and 5X) with χ2 values of 1.11, 0.71, 1.59, and 0.05, and corresponding *P* values of 0.29, 0.42, 0.21, and 0.63, respectively. These results suggest that a single gene controls resistance to these four strains. The segregation ratios for seven strains (3A, 2B, 5C, 5G, 8J, 5L, and 3O) were 1R:3S, with χ² values of 2.88, 0.07, 0.01, 0.46, 0.25, 0.04, and 2.12, and corresponding *P* values of 0.09, 0.82, 0.90, 0.50, 0.64, 0.86, and 0.16, respectively. These results suggest that two genes with complementary action control resistance to these seven strains.

**Table 1:** Clubroot resistance in the parental lines (DH16516, ECD10) and BC_1_S_1_ families inoculated with 11 strains of *Plasmodiophora brassicae* based on the disease severity index (DSI) of each family.

Strain	DSIs		No. of BC_1_S_1_ families			*P* value of ratio			Inheritance
	ECD10	DH16516	Total	R (DSI ≤ 60%)	S (DSI > 60%)	χ2 value	1:1	1:3	
3A	3.3	100.0	92	16	76	2.88	–	0.09	2 complementary genes
2B	0.0	100.0	92	22	70	0.07	–	0.82	2 complementary genes
5C	13.9	100.0	91	23	68	0.01	–	0.90	2 complementary genes
3D	0.0	100.0	92	41	51	1.11	0.29	–	Single gene
8E	0.0	100.0	92	42	50	0.71	0.42	–	Single gene
5G	8.3	97.2	91	20	71	0.46	–	0.50	2 complementary genes
8J	0.0	100.0	92	21	71	0.25	–	0.64	2 complementary genes
5L	0.0	97.2	91	22	69	0.04	–	0.86	2 complementary genes
3O	19.4	94.4	92	17	75	2.12	–	0.16	2 complementary genes
8P	0.0	100.0	92	40	52	1.59	0.21	–	Single gene
5X	1.5	100.0	92	45	47	0.05	0.63	–	Single gene

### Alignment of short read into the *B. napus* genome ‘ZS11’ v0

The genome sequence of the Chinese canola cultivar ‘ZS11’ was the completest *B. napus* genome available to this project when we initiated the study. GBS was carried out on the BC_1_ individuals for SNP identification, genetic mapping, and QTL mapping using ‘ZS11’ genome. A total of 1,252.7 million (M) short reads were obtained across two combined replicates each of the parental lines and 92 BC_1_ plants (**Supplementary Table S1**). Approximately 13.4 and 29.0 M short reads were obtained from the combined parents ECD10 and DH16516, respectively. Of these, 12.9 M (96.5%) and 27.9 M (96.1%) reads were assembled to the ‘ZS11’ genome. Totally, 1,210.3 M short read sequences were obtained from 92 BC_1_ plants, ranging from 1.9 to 20.5 M/plant, with an average of 13.2 M/plant (**Supplementary Table S1**). The average read assembled to the *B. napus* genome was 12.5 M for each BC_1_ plant with a range from 1.2 to 19.8 M, corresponding to an alignment percentage between 64.8% and 96.7% (**Supplementary Table S1**).

### SNP identification and polymorphic SNP selection

A total of 1,307,115 alleles and 14,055 SNPs were identified across 92 BC_1_ plants (**Supplementary Table S2**). The average frequencies of the major and minor alleles at each site were 0.7 and 0.3, with ranges of 0.5–0.9 and 0.1–0.5, respectively (**Supplementary Table S2**). The 14,055 SNPs identified from the 92 BC_1_ plants were distributed across the 19 chromosomes of the *B. napus* ‘ZS11’ genome. On average, there were 739.7 SNPs per chromosome, with a range of 316 to 2,151 SNPs. The SNP distribution correlated with the length of the reference genome sequence (*R²* = 0.55) (**Supplementary Table S3**). Following initial filtering, 5,722 polymorphic SNP sites were mapped to the 19 chromosomes. The number of these polymorphic SNPs correlated with chromosome size (*R²* = 0.58) (**Supplementary Table S3**).

### Construction of genetic map

**Figure 3:**
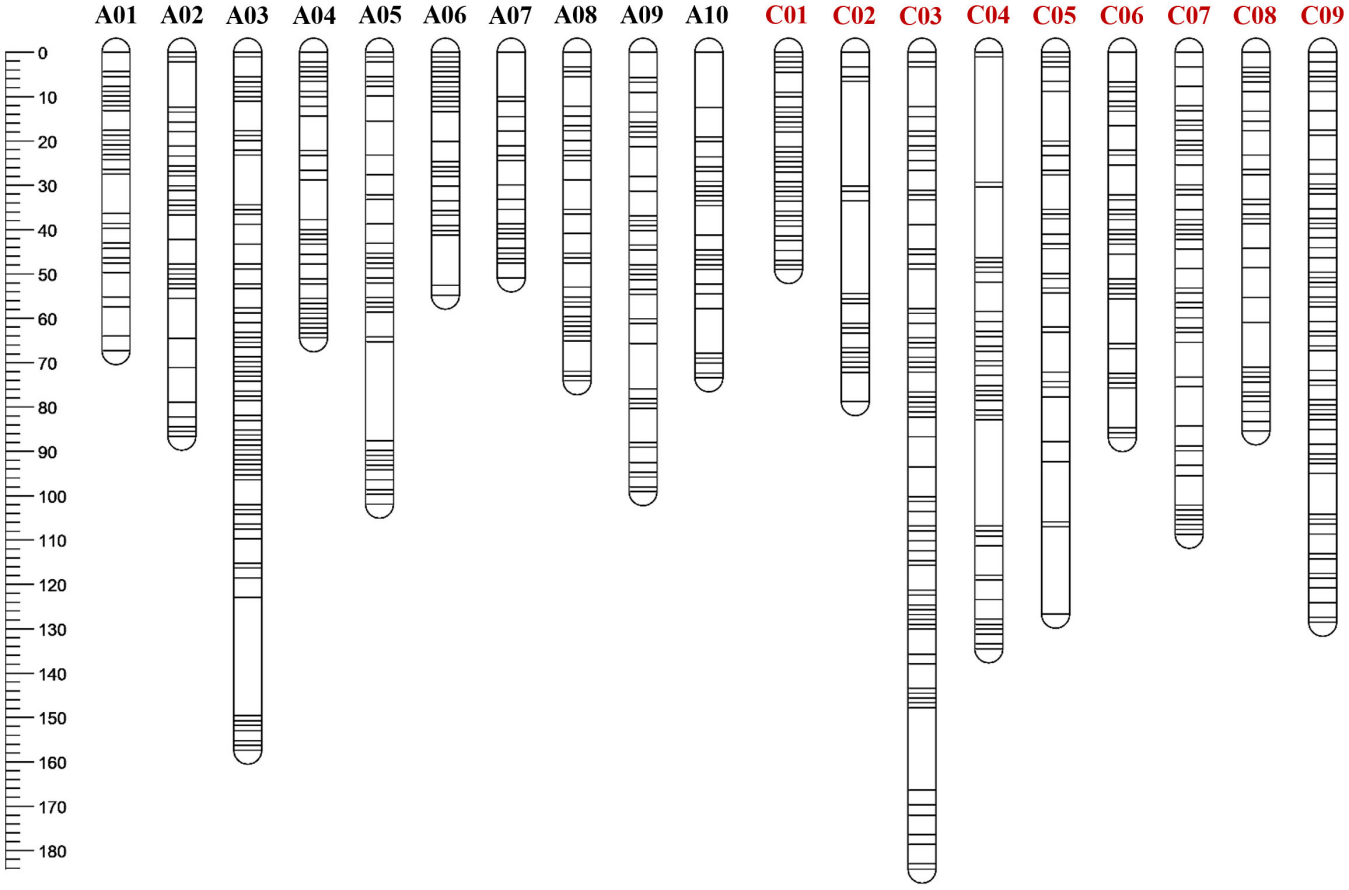
The genetic map of *Brassica napus* containing 1,049 polymorphic SNPs extracted using *Brassica napus* reference genome of a Chinese canola cultivar ‘ZS11’.

A total of 941 and 3,732 SNPs were removed by the JoinMap and BIN functions of QTL IciMapping respectively (**Supplementary Table S3**). A genetic or linkage map was constructed consisting of 19 linkage groups, spanning a total of 1,812.6 cM, derived from 1,049 non-redundant polymorphic SNP sites (**Figure 3**). The average number of SNPs identified on each chromosome was 55.2, in a range of 28 to 98. Chromosome lengths varied from 49 to 184.2 cM, with 95.4 cM on average. The genetic distance between two SNPs per chromosome ranged from 0.8 to 2.9 cM, with an interval of 1.8 cM on average in the *B. napus* genome (**Supplementary Table S3**).

### Identification of QTL for resistance to clubroot

**Figure 4:**
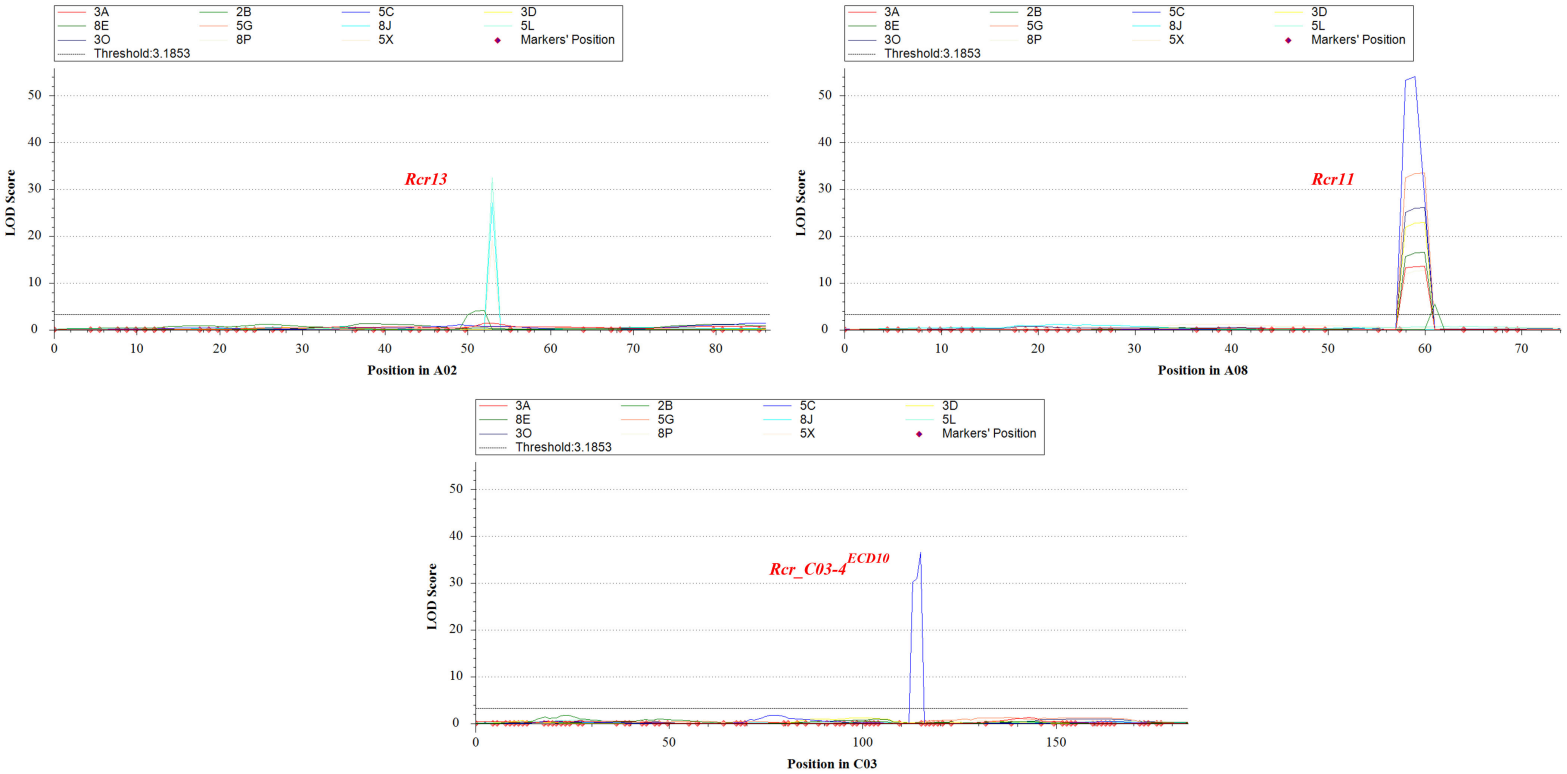
Three significant QTL; *Rcr13*, *Rcr11* and *Rcr_C03-4^ECD10^* identified on chromosome A02, A08 and C03 for resistance to clubroot from *Brassica napus* rutabaga ECD10.

Identification of QTL for clubroot resistance was carried out using the genetic map as shown in **Figure 3**, correlating to % DSI values with resistance to the 11 strains described in **Table 1**. Two QTL located on chromosomes A02 and A08 respectively were identified for resistance to multiple strains, while one QTL on chromosome C03 was specific to one strain (5C) only (**Figure 4**). The QTL on chromosome A02, designated *Rcr13*, confers resistance to strains 2B, 8J, 5L, and 5X. It was located within the 23.65–28.32 Mb region of the ‘ZS11’ reference genome sequence, with peak markers at ZS11_A02_23,656,591 and ZS11_A02_28,328,467. The QTL exhibited logarithm of odds (LOD) scores ranging from 4.1 to 32.5, PVE between 14.2% and 81.7%, Add ranging from 18.0 to 48.2, and a confidence interval (CI) between 2.4 and 2.6 (**Table 2**). On chromosome A08, the QTL designated *Rcr11* was detected for resistance to 8 strains (3A, 2B, 5C, 3D, 8E, 5G, 3O, and 8P). This QTL was located within the 14.99–19.00 Mb region of the ‘ZS11’ genome, with peak markers at ZS11_A08_14,991,569 and ZS11_A08_19,004,127. *Rcr11* exhibited LOD scores ranging from 5.6 to 54.1, PVE between 20.2% and 83.2%, Add between 21.3 and 74.5, and CI between 0.8 to 1.9. Additionally, a QTL on chromosome C03, designated *Rcr_C03-4^ECD10^*, was detected for resistance to the stain 5C. The QTL was located within the 17.91–18.09 Mb region of the ‘ZS11’ genome sequence, with peak markers at ZS11_C03_17,919,068 and ZS11_C03_18,092,379. It exhibited a LOD score of 36.6, explained 21.5% of the PVE, had an Add of 45.2, and a CI of 1.0 Mb. The Add values for all three identified QTL were positive, demonstrating that the resistance alleles were contributed by the resistant parent ECD10 (**Table 2**).

**Table 2:** Identification of QTL for resistance to clubroot in the *Brassica napus* rutabaga cultivar ECD10 using the *Brassica napus* reference genome sequence ‘ZS11’.

Chromosome/QTL	Strain	Position	Left Marker	Right Marker	LOD	PVE (%)	Add	Left CI	Right CI	Interval (Mb)
A02/*Rcr13*	2B	52	ZS11_A02_23,656,591	ZS11_A02_26,234,443	4.1	14.2	18.0	49.5	52.5	2.6
	8J	53	ZS11_A02_25,884,980	ZS11_A02_28,328,467	27.1	72.6	41.8	52.5	53.5	2.4
	5L	53	ZS11_A02_25,884,980	ZS11_A02_28,328,467	32.5	81.7	43.7	52.5	53.5	2.4
	5X	53	ZS11_A02_25,884,980	ZS11_A02_28,328,467	19.0	57.2	48.2	52.5	53.5	2.4
A08/*Rcr11*	3A	60	ZS11_A08_17,062,456	ZS11_A08_16,283,079	13.5	51.2	25.7	57.5	60.5	0.8
	2B	61	ZS11_A08_16,705,471	ZS11_A08_14,991,569	5.6	20.2	21.3	60.5	61.5	1.7
	5C	59	ZS11_A08_19,004,127	ZS11_A08_17,062,456	54.1	58.2	74.5	57.5	59.5	1.9
	3D	60	ZS11_A08_17,062,456	ZS11_A08_16,283,079	22.9	72.3	51.2	57.5	60.5	0.8
	8E	60	ZS11_A08_17,062,456	ZS11_A08_16,283,079	16.5	58.7	46.0	57.5	60.5	0.8
	5G	60	ZS11_A08_17,062,456	ZS11_A08_16,283,079	33.5	83.2	39.0	57.5	60.5	0.8
	3O	60	ZS11_A08_17,062,456	ZS11_A08_16,283,079	26.1	75.7	36.1	57.5	60.5	0.8
	8P	60	ZS11_A08_17,062,456	ZS11_A08_16,283,079	12.7	50.6	40.2	57.5	60.5	0.8
C03/*Rcr_C03-4^ECD10^*	5C	115	ZS11_C03_18,092,379	ZS11_C03_17,919,068	36.6	21.5	45.2	114.5	115.5	1.0

LOD, logarithm of odds; PVE, phenotypic variation explained; Add, additive effects; CI, confidence interval.

### Identification of potential candidate genes for the QTL

Genes located in the intervals of *Rcr11*, *Rcr13*, and *Rcr_C03-4^ECD10^* were identified using the coding sequence of the ‘ZS11’ reference genome through Blast2GO. Candidate genes that encode proteins for disease resistance were found (**Supplementary Tables S4**-**S8**).

*Rcr13* was localized on chromosome A02 within the 23.65–28.32 Mb region. There were 384 genes identified in the region (**Supplementary Table S4**), including 31 genes encoding disease resistance-related proteins (**Supplementary Table S7**). Notably, nine genes (*BnaA02G0287000ZS*, *BnaA02G0291600ZS*, *BnaA02G0291700ZS*, *BnaA02G0291800ZS*, *BnaA02G0291900ZS*, *BnaA02G0292000ZS*, *BnaA02G0292100ZS*, *BnaA02G0299700ZS*, and *BnaA02G0299900ZS*) encoded TIR-NBS-LRR (TNL) class proteins, which are homologous to seven TNL-encoding Arabidopsis genes (*AT5G40910.1*, *AT2G17060.1*, *AT5G45240.1*, *AT5G45250.1*, *AT5G45260.2*, *AT5G22690.1*, and *AT5G40060.1*) (**Table 3**).

**Table 3:** Disease resistance genes in the intervals of *Rcr13*, *Rcr11* and *Rcr_C03-4^ECD10^* of chromosomes A02, A08 and C03 in the Chinese canola cultivar ‘ZS11’ genome and the gene homologs in Arabidopsis.

QTL (Corresponding strains)	Gene name	Start	End	Description from Blast2Go	Homolog in Arabidopsis	Description from TAIR
*Rcr13* (23.6–28.3 Mb) (2B, 8J, 5L, 5X)	*BnaA02T0287000ZS*	25,444,181	25,446,241	disease resistance protein (TIR-NBS-LRR class)	*AT5G40910.1*	disease resistance protein (TIR-NBS-LRR class)
	*BnaA02T0291600ZS*	25,953,735	25,959,215	disease resistance-like protein CSA1	*AT2G17060.1*	disease resistance protein (TIR-NBS-LRR class)
	*BnaA02T0291700ZS*	25,976,806	25,980,115	disease resistance protein (TIR-NBS-LRR class)	*AT5G45240.1*	disease resistance protein (TIR-NBS-LRR class)
	*BnaA02T0291800ZS*	25,980,218	25,980,892	disease resistance protein LAZ5-like	*AT5G45240.1*	disease resistance protein (TIR-NBS-LRR class)
	*BnaA02T0291900ZS*	25,994,280	26,005,347	disease resistance protein RPS4-like	*AT5G45250.1*	disease resistance protein (TIR-NBS-LRR class)
	*BnaA02T0292000ZS*	26,026,172	26,032,673	disease resistance protein RRS1	*AT5G45260.2*	confers resistance to *Ralstonia solanacearum*. Similar to NBLS-TIR resistance genes
	*BnaA02T0292100ZS*	26,034,277	26,038,253	disease resistance protein (TIR-NBS-LRR class)	*AT5G45240.1*	disease resistance protein (TIR-NBS-LRR class)
	*BnaA02T0299700ZS*	26,883,052	26,883,390	disease resistance protein RPS6-like	*AT5G22690.1*	disease resistance protein (TIR-NBS-LRR class)
	*BnaA02T0299900ZS*	26,910,001	26,914,245	disease resistance protein RPS6-like	*AT5G40060.1*	disease resistance protein (NBS-LRR class)
*Rcr11* (14.9–19.0 Mb) (3A, 2B, 5C, 3D, 8E, 5G, 3O and 8P)	*BnaA08T0114900ZS*	17,221,061	17,225,029	inactive disease resistance protein RPS4-like	*AT4G19510.2*	disease resistance protein (TIR-NBS-LRR class)
	*BnaA08T0117100ZS*	17,342,502	17,343,071	disease resistance protein TAO1-like	*AT5G11250.1*	encodes an atypical TIR-NBS-LRR protein
	*BnaA08T0141000ZS*	19,001,038	19,002,195	Toll-Interleukin-Resistance (TIR) domain family protein	*AT1G57850.2*	Toll-Interleukin-Resistance (TIR) domain family protein
*Rcr_C03-4^ECD10^* (17.9–18.0 Mb) (5C)	*BnaC03T0281900ZS*	18,038,047	18,041,472	probably inactive leucine-rich repeat receptor-like protein kinase	*AT2G25790.1*	Leucine-rich receptor-like protein kinase family protein

*Rcr11* was located on chromosome A08 within the 14.99–19.00 Mb region. In the region, 542 genes were identified (**Supplementary Table S5**), including five genes (*BnaA08G0102200ZS*, *BnaA08G0108700ZS*, *BnaA08G0114900ZS*, *BnaA08G0117100ZS*, and *BnaA08G0141000ZS*) that encode disease resistance-related proteins (**Supplementary Table S7**). Among these genes, three (*BnaA08G0114900ZS*, *BnaA08G0117100ZS*, and *BnaA08G0141000ZS*) were homologous to the genes *AT4G19510.2*, *AT5G11250.1* and *AT1G57850.2* in Arabidopsis, which encode TNL class proteins, suggesting that they are probably candidate genes for *Rcr11* (**Table 3**).

*Rcr_C03-4^ECD10^* was identified on chromosome C03 within the 17.91–18.09 Mb region, showing resistance to strain 5C. Eighteen genes were identified in the region (**Supplementary Table S6**). One gene, *BnaC03G0281900ZS*, was homologous to *AT2G25790.1*, which encodes a leucine-rich repeat receptor-like protein kinase and is a probable candidate gene for *Rcr_C03-4^ECD10^* (**Table 3**; **Supplementary Table S7**).

### Search for syntenic regions for *Rcr13* and *Rcr11* of disease resistance-related genes identified from *B. napus* ‘ZS11’ in Chinese cabbage (*B. rapa*) ‘Chiifu’

*B. rapa* serves as a major source for identifying CR genes or QTL in *Brassica* crops. In the current study, the BC_1_/BC_1_S_1_ population in *B. napus* was used for genetic mapping, and two major QTL *Rcr13* and *Rcr11*, which confer resistance to multiple strains, were identified on chromosomes A02 and A08 respectively. Previously identified genes, *Rcr8* and *Rcr3/Rcr9*, were found in *B. rapa* turnip (Yu et al., 2017; Karim et al., 2020; Yu et al., 2021) on chromosomes A02 and A08. The newer version of the *B. rapa* reference genome, ‘Chiifu’ v3.0 (Zhang et al., 2018), was used to compare and establish synteny in the target regions of the QTL.

In this study, the flanking region of *Rcr13* (23.65–28.32 Mb) in the *B. napus* reference genome sequence ‘ZS11’ was found to be homologous to the region on chromosome A02 (19.41–23.86 Mb) of *B. rapa* ‘Chiifu’ v3.0. Among the 31 disease resistance-related genes identified in the flanking region of *Rcr13* in the *B. napus* reference genome, 21 genes showed homology with the A02 region (21.05–23.17 Mb) of ‘Chiifu’ v3.0. The previously identified CR QTL, *Rcr8* (A2: 22.50–26.34 Mb), was located within the region of *Rcr13* on A02 of ‘Chiifu’ v3.0 (**Supplementary Table S8**).

Similarly, the flanking region of *Rcr11* (14.99–19.00 Mb) in the *B. napus* reference genome sequence ‘ZS11’ was homologous to the region on chromosome A08 (9.11–13.45 Mb) of *B. rapa* ‘Chiifu’ v3.0. Among the five disease resistance genes identified in the flanking region of *Rcr11* in the *B. napus* reference genome, two genes showed homology with the A08 region (11.17–11.90 Mb) of ‘Chiifu’ v3.0. The previously identified major CR genes, *CRs* (A08: 11.34–12.16 Mb) and *Rcr3*/*Rcr9* (11.38–12.65 Mb) were located within the interval of *Rcr11* on A08 of ‘Chiifu’ v3.0 (**Supplementary Table S8**).

### Search for the syntenic region of the *Rcr_C03-4^ECD10^* disease resistance-related gene identified from *Brassica napus* ‘ZS11’ on chromosome C03 of the *Brassica oleracea* reference genome ‘D134’

Recently, three QTL on chromosome C03 were identified in the *B. oleracea* cultivar ECD11 (Karim and Yu, 2023). In this study, the region of *Rcr_C03–4 ^ECD10^* (17.91–18.09 Mb) in the *B. napus* reference genome sequence ‘ZS11’ was found to be homologous to the region on chromosome C03 (56.34–56.53 Mb) of *B. oleracea* ‘D134’ v3.0. The putative disease resistance gene *BnaC03G0281900ZS* for *Rcr_C03–4 ^ECD10^* in the *B. napus* reference genome sequence ‘ZS11’ showed homology to *Boc03g04362* (56.40–56.41 Mb) of *B. oleracea* ‘D134’ v3.0 (**Supplementary Table S9**). In comparison, the previously identified C03 CR QTL of ECD11, namely *Rcr_C03-1* (C03: 9.21–17.06 Mb), *Rcr_C03-2* (C03: 5.85–10.26 Mb), and *Rcr_C03-3* (C03: 35.22–36.28 Mb), were located in different regions of ‘D134’ v3.0 (Karim and Yu, 2023).

### Search for syntenic regions of disease resistance genes across different *Brassica napus* reference genomes

In the current study, the ‘ZS11’ reference genome was used for read assembly, and a comparison was conducted using eight other *Brassica napus* reference genomes (**Supplementary Table S10**). ‘ZS11’ contained the highest number of predicted disease resistance genes (2,708), followed by ‘No2127’ (2,643), ‘Tapidor’ (2,578), ‘Quinta’ (2,510), ‘Zheyou7’ (2,509), ‘Shengli’ (2,481), ‘Darmor-bzh’ (2,468), ‘Westar’ (2,395), and ‘Gangan’ (29). The flanking regions of the identified QTL were compared across these reference genomes: *Rcr13* on chromosome A02, *Rcr11* on A08, and *Rcr_C03-4^ECD10^* on C03. For *Rcr13*, the highest number of resistance genes was identified in ‘Shengli’ (38), followed by ‘Gangan’ (24), ‘No2127’ (24), ‘Zheyou7’ (24), ‘Tapidor’ (23), ‘Quinta’ (22), ‘ZS11’ (22), and ‘Westar’ (21). For *Rcr11*, the highest number was found in ‘Shengli’ (39), followed by ‘Zheyou7’ (36), ‘ZS11’ (21), ‘No2127’ (19), ‘Gangan’ (17), ‘Quinta’ (16), ‘Tapidor’ (14), and ‘Westar’ (6). For *Rcr_C03-4^ECD10^*, the highest number of resistance genes was identified in ‘No2127’ (28), followed by ‘Zheyou7’ (24), ‘Quinta’ (2), ‘Gangan’ (1), ‘Shengli’ (1), ‘Westar’ (1), and ‘ZS11’ (1).

### Differential reactions of breeding lines carrying *Rcr13* vs *Rcr8* as well as *Rcr11* vs *Rcr3/Rcr9*

**Figure 5:**
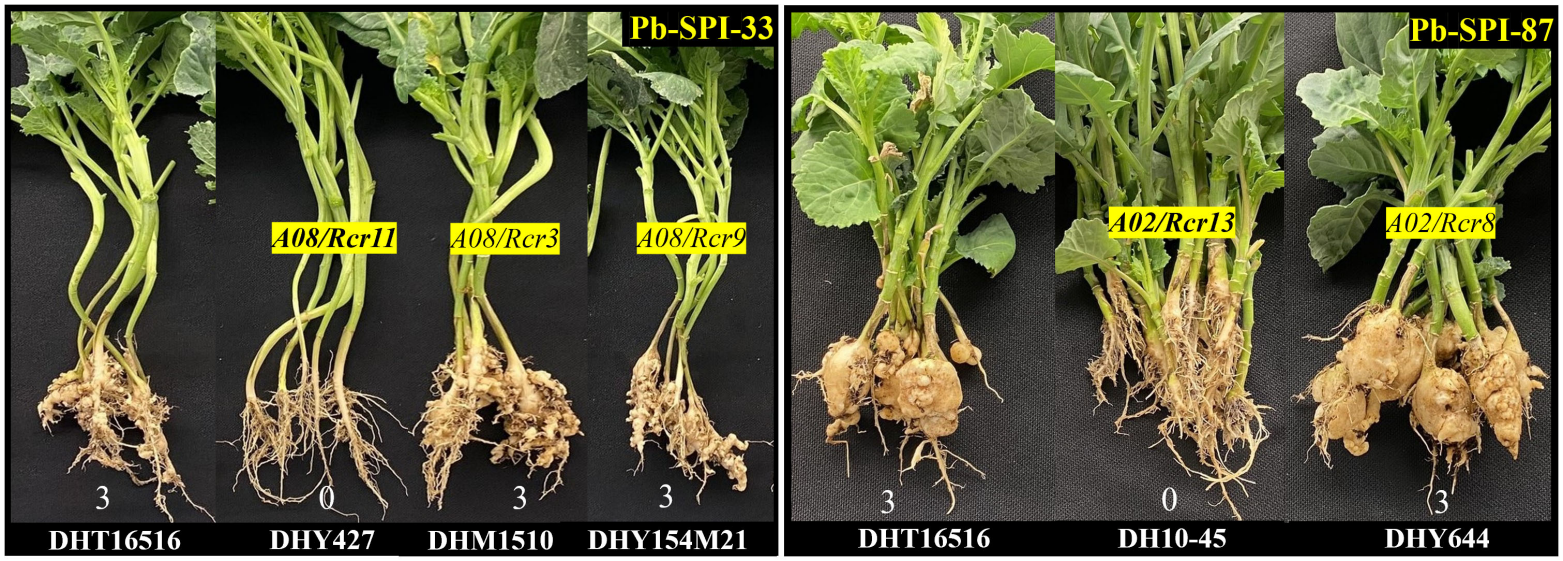
Differential reactions of the identified genes *Rcr3*, *Rcr8*, *Rcr9*, *Rcr11*, and *Rcr13* introgressed into spring-type canola, assessed using purified *Plasmodiophora brassicae* isolates, Pb-SPI-33 and Pb-SPI-87. Susceptible parental line, DHT16516, served as control, exhibiting highly susceptible reaction with both isolates. The lines DHY427 (*Rcr11*), DHM1510 (*Rcr3*), DHY154M21 (*Rcr9*), DH10-45 (*Rcr13*), and DHY644 (*Rcr8*) demonstrated isolate-specific resistance patterns, confirming the distinct resistance mechanisms of these genes.

*Rcr13* and *Rcr11* were identified on chromosomes A02 and A08 respectively in this study, Interestingly, CR genes *Rcr8* and *Rcr3/Rcr9* resistant to Canadian strains of *P. brassicae* were previously mapped into chromosomes A02 and A08 (Yu et al., 2017, 2021; Rahaman, 2022). Five *B. napus* breeding lines DHY644, DH10-45, DHY427, DHM1510, and DHY154M21, carrying the resistance genes *Rcr8, Rcr13, Rcr11, Rcr3* and *Rcr9* respectively, were evaluated for their differential reactions using 4 purified *P. brassicae* isolates Pb-SPI-33, Pb-SPI-87, Pb-SPI-110 and Pb-SPI-151 (**Table 4**). The susceptible parental line DH16516 displayed susceptibility to all four isolates. Two isolates Pb-SPI-87 and Pb-SPI-110 could differentiate disease reactions of the breeding line carrying *Rcr13* (DH10-45) from that of *Rcr8* (DHY644), while the other two isolates Pb-SPI-87 and Pb-SPI-151 showed clear differential reactions from the breeding line carrying *Rcr11* (DHY427) with those of *Rcr3* and *Rcr9* (DHM1510 and DHY154M21) (**Table 4**). *Rcr13*-containing line DH10**–**45 exhibited a high level of resistance to Pb-SPI-87 and Pb-SPI-110 (0% DSI), whereas the *Rcr8*-containing line DHY644 was highly susceptible (100% DSI) (**Figure 5**). The line containing *Rcr11* (DHY427) demonstrated resistance to both Pb-SPI-33 and Pb-SPI-151, whereas the lines containing *Rcr3* and *Rcr9* (DHM1510 and DHY154M21, respectively) were susceptible to these isolates (**Table 4**).

**Table 4:** Disease severity index of the *B. napus* breeding lines carrying *Rcr13* and *Rcr8* on chromosome A02 and *Rcr11* and *Rcr3/Rcr9* on chromosome A08 inoculated with the purified *Plasmodiophora brassicae* isolates.

Breeding line	DH16516	DH10-45	DHY644	DHY427	DHM1510	DHY154M21
Chrom./Gene name	no CR gene	A02/*Rcr13*	A02/*Rcr8*	A08/*Rcr11*	A08/*Rcr3*	A08/*Rcr9*
Pb-SPI-33	100	–	–	0	100	100
Pb-SPI-87	100	0	100	–	–	–
Pb-SPI-110	100	0	100	–	–	.
Pb-SPI-151	100	–	–	0	100	100

## Discussion

In Canada, the need for adopting new sources of clubroot resistance in canola has become urgent because of the rapid emergence of new strains and the breakdown of the ‘Mendel’ CR gene on chromosome A03 in the first generation of CR cultivars. Using CR genes from *B. napus* sources is prioritized in canola breeding to avoid unintended linkage associations with traits from other progenitor species. However, clubroot resistance within *B. napus* itself is not common. The ‘Mendel’ clubroot resistance was widely used in canola breeding in Europe and Canada until it broke down in Canada in 2013. Several research groups have investigated clubroot resistance in different brassica species, including *B. rapa* (Chu et al., 2014; Gao et al., 2014; Yu et al., 2016; Huang et al., 2017; Karim et al., 2020), *B. oleracea* (Farid et al., 2020; Dakouri et al., 2018; Karim and Yu, 2023), and *B. napus* (Hasan and Rahman, 2016; Zhang et al., 2016; Fredua-Agyeman and Rahman, 2016, 2021; Dakouri et al., 2021; Yu et al., 2021). However, many studies focusing on identifying clubroot resistance in *B. napus* have mainly involved older or a limited number of new Canadian pathotypes. For instance, a single major gene on A08 was reported to confer resistance against five older Canadian pathotypes (2, 3, 5, 6, and 8) from *B. napus* subsp. *napobrassica* ‘Brookfield’ (Hasan and Rahman, 2016). Another study identified a major gene on A03 that provides resistance against pathotype 3 from a spring canola inbred line (12-3) (Zhang et al., 2016), which inherited its clubroot resistance from the European fodder turnip ECD04, a clubroot resistance donor line for the oilseed rape cultivar ‘Mendel’. Additionally, a major gene on chromosome A03 conferring resistance against pathotype 3 was reported from *B. napus* ‘Mendel’ (Fredua-Agyeman and Rahman, 2016). Furthermore, the *Rcr3^Mendel^* gene on A08 was shown to confer resistance against strains 3H, 5C, 3D, and 8J (Rahaman, 2022). Two major QTL, *Rcr10^ECD01^* and *Rcr9^ECD01^*, were found for resistance to strains 3A, 3D, 3H and 5X on chromosomes A03 and A08 in the introgressed *B. napus* lines from *B. rapa* ECD01 (Yu et al., 2021). ECD10 has been noted for its resistance to all 17 pathotypes in the CCD set (Strelkov et al., 2018), making it highly attractive for canola breeding. Therefore, genetic mapping and identification of CR genes from ECD10 against major Canadian pathotypes aggressive to the ‘Mendel’ source have become essential tasks in current research efforts.

In a recent study, eight high-quality reference genomes representing different ecotypes of *B. napus* were published to enhance the understanding of the genome structure and genetic basis for morphotype differentiation (Song et al., 2020). One of these reference genomes, the ‘ZS11’ genome, was assembled using integrated PacBio, Hi-C, and BioNano data, resulting in well-anchored chromosomes and a comprehensive set of annotated genes. This ‘ZS11’ genome served as the foundation for aligning GBS short reads, conducting genetic mapping, and performing biparental QTL mapping in the present study. Typically, the number of SNP sites per chromosome correlates with chromosome size (Yu et al., 2016). Similarly, our study revealed a correlation between the numbers of total SNPs and polymorphic SNPs identified in the population with those in the reference genome ‘ZS11’ (*R2 =* 0.55, 0.58). The construction of a genetic map is essential for QTL analysis, where the map length depends on factors such as population size, type, number of polymorphic variants, and the quality of the reference genome. In this study, we successfully constructed a genetic map spanning 1,812.6 cM using 1,049 polymorphic variants across 92 BC_1_ plants.

In this study, two major QTL on the A-genome, *Rcr11* and *Rcr13*, and one minor QTL on the C-genome, *Rcr_C03-4^ECD10^*, were identified, conferring resistance to 11 strains. Major QTL are defined as those contributing more than 10% of the PVE and conferring resistance to multiple strains (Wang et al., 2019; Karim and Yu, 2023). *Rcr13* and *Rcr11* showed substantial contributions to PVE, explaining between 14.2% to 81.7% and 20.2% to 83.2% of the phenotypic variation, respectively. They conferred resistance to multiple strains: *Rcr13* to 2B, 8J, 5L, and 5X, and *Rcr11* to 3A, 2B, 5C, 3D, 8E, 5G, 3O, and 8P. Therefore, *Rcr11* and *Rcr13* are considered major QTL due to their significant contributions to resistance across multiple strains. On the other hand, *Rcr_C03-4^ECD10^* in the C-genome contributed to resistance against a single strain, 5C, with a PVE of 21.5%. Consequently, *Rcr_C03-4^ECD10^* is classified as a minor QTL because it provides resistance to a narrower spectrum of strains than *Rcr11* and *Rcr13*. Therefore, *Rcr11* and *Rcr13* are major QTL due to their high PVE and broad-spectrum resistance, whereas *Rcr_C03-4^ECD10^* is a minor QTL with narrower specificity in pathotype resistance.

The parental lines used in this study, ECD10 (R) and DH16516 (S), displayed contrasting responses to all the 11 strains tested: 3A, 2B, 5C, 3D, 8E, 5G, 8J, 5L, 3O, 8P, and 5X. The segregation ratios observed among the 92 BC_1_S_1_ families indicated that different genetic control mechanisms underlie resistance to these strains in ECD10. For four strains (3D, 8E, 8P, and 5X), the segregation ratio of 1R:1S suggested that resistance in ECD10 might be governed by a single resistance gene for each of these strains. This observation is consistent with the identification of QTL *Rcr11* for 3D, 8E, and 8P, and *Rcr13* for 5X, indicating that each QTL corresponds to a single gene controlling resistance. However, for seven other strains (3A, 2B, 5C, 5G, 8J, 5L, and 3O), the segregation ratio of 1R:3S suggested that resistance in ECD10 is likely controlled by two genes exhibiting complementary action. QTL mapping revealed two QTL, *Rcr11* and *Rcr13* for 2B, and *Rcr11* and *Rcr_C03-4^ECD10^* for 5C, which are consistent with these observations, suggesting that two genes are involved in complementary action for resistance to these strains. Interestingly, for strains 3A, 5G, and 3O, only one QTL, *Rcr11*, was identified, despite the segregation ratio suggesting that the two genes have complementary effects. Similarly, for strains 8J and 5L, only one QTL, *Rcr13*, was identified, contrary to the expected segregation ratio indicating two genes. The discrepancy between the expected segregation ratios and the identified QTL suggests the potential loss of CR genes in the current mapping population. Further investigations are underway to identify the lost CR genes in the current mapping population.

All three identified QTL (*Rcr11*, *Rcr13*, and *Rcr_C03-4^ECD10^*) were found to be homologous to several disease resistance proteins. There were nine potential candidate genes (*BnaA02G0287000ZS*, *BnaA02G0291600ZS*, *BnaA02G0291700ZS*, *BnaA02G0291800ZS*, *BnaA02G0291900ZS*, *BnaA02G0292000ZS*, *BnaA02G0292100ZS*, *BnaA02G0299700ZS*, and *BnaA02G0299900ZS*) in the flanking region of *Rcr13*, all of which are homologous to TNL genes in Arabidopsis. In the interval of *Rcr11*, five genes (*BnaA08G0102200ZS*, *BnaA08G0108700ZS*, *BnaA08G0114900ZS*, *BnaA08G0117100ZS*, and *BnaA08G0141000ZS*) encode disease resistance proteins, and three of these genes (*BnaA08G0114900ZS*, *BnaA08G0117100ZS*, and *BnaA08G0141000ZS*) were identified as potential candidate genes homologous to Arabidopsis TNL genes. There was one potential candidate gene (*BnaC03G0281900ZS*) encoding a leucine-rich repeat receptor-like protein kinase identified in the flanking region of *Rcr_C03-4^ECD10^*. In total, 12 genes encoding TNL class proteins were identified for *Rcr11* and *Rcr13*. Notably, TNL class proteins involved in clubroot resistance such as CR genes *CRa*, *Crr1a*, and *CRb* and *Rcr1* have previously been cloned (Ueno et al., 2012; Hatakeyama et al., 2013, 2017; Hu et al., 2024).

Previously, QTL conferring resistance to different strains were identified from various *Brassica* resistance sources on chromosomes A02, A08, and C03 (Suwabe et al., 2003; Sakamoto et al., 2008; Hatakeyama et al., 2013; Yu et al., 2017; Laila et al., 2019; Zhu et al., 2019; Farid et al., 2020; Karim et al., 2020; Karim and Yu, 2023). The QTL identified in this study along with candidate CR genes were compared with the more recent version of the *B. rapa* reference genome ‘Chiifu’ v3.0 (Zhang et al., 2018) to establish relationships between the current and previously identified QTL. For *Rcr13*, nine candidates potentially for clubroot resistance of *B. napus* were identified, homologous to seven *B. rapa* genes, namely, *BraA02g031160.3C* (21,155,765–21,156,434), *BraA02g031640.3C* (21,553,166–21,556,336), *BraA02g031710.3C* (21,630,856–21,633,310), *BraA02g031640.3C* (21,553,166–21,556,336), *BraA02g031610.3C* (21,543,465–21,545,005), *BraA02g032880.3C* (22,615,923–22,619,152), and *BraA02g032920.3C* (22,653,678–22,656,240). Two clusters of TNL genes were identified for *Rcr13*: one cluster (21.15–21.63 Mb) of novel CR genes, and another cluster (22.61–22.65 Mb), which includes *BraA02g032880.3C* (22,615,923–22,619,152) and *BraA02g032920.3C* (22,653,678–22,656,240). The second cluster was located in the interval of the major CR gene *Rcr8* (22.50–26.34 Mb) (Yu et al., 2017). *Rcr13* has been confirmed as a putatively novel QTL, distinct from *Rcr8* as breeding lines carrying *Rcr8* and *Rcr13* exhibited different specificity against clubroot isolates. The differential reactions observed among the introgressed lines highlight distinct resistance mechanisms. The *Rcr13*-containing line DH10–45 demonstrated resistance to Pb-SPI-87 and Pb-SPI-110 but the *Rcr8*-containing line DHY644 was susceptible to the isolates. Despite both genes being located on chromosome A02, this finding confirms that *Rcr8* and *Rcr13* are distinct genes. The TNL genes from the first cluster may be more likely candidate genes for *Rcr13*.

Three genes (*BnaA08G0114900ZS*, *BnaA08G0117100ZS*, and *BnaA08G0141000ZS*) of *B. napus* were identified as candidates for *Rcr11*, homologous to the genes that encodes TNL class proteins in Arabidopsis. By comparing the A08 genes ‘ZS11’ and ‘Chiifu’ v3.0, two candidate genes for *Rcr11*, *BnaA08G0114900ZS* and *BnaA08G0117100ZS*, were found to be homologous in the interval between 11.18–11.90 Mb of the A08 chromosome of ‘Chiifu’ v3.0, an overlapping region of *CRs* (11.34–12.16 Mb) and *Rcr3/Rcr9* (11.38–12.65 Mb) (Karim et al., 2020; Yu et al., 2017, 2021). *Rcr11* has been confirmed as a putatively novel QTL, different from *Rcr3/Rcr9*, based on testing results with the *B. napus* lines containing single CR genes *Rcr3*, *Rcr9* and *Rcr11* developed in our group. To differentiate the loci *Rcr11*, *Rcr3*, and *Rcr9*, the isolates Pb-SPI-33 and Pb-SPI-151 were used. Lines containing the *Rcr3* and *Rcr9* loci (DHM1510 and DHY154M21, respectively) exhibited susceptibility to both isolates. In contrast, the *Rcr11*-containing line (DHY427) demonstrated resistance to both Pb-SPI-33 and Pb-SPI-151. This indicates that either of these purified isolates can effectively distinguish between the resistance conferred by *Rcr11* with *Rcr3* and *Rcr9*. Despite all three genes being located on chromosome A08, this finding confirms that *Rcr11* is a different CR gene from *Rcr3* or *Rcr9*. Therefore, *BnaA08G0141000ZS* is the most likely candidate gene for *Rcr11*. A08 is considered to have a cluster of CR genes (Hasan and Rahman, 2016), so different CR genes from the same flanking region could contribute to clubroot resistance against different strains.

For *Rcr_C03-4^ECD10^*, a probable disease resistance gene, *BnaC03G0281900ZS*, was identified as homologous to *Boc03g04362* (56.40–56.41 Mb) of *B. oleracea* ‘D134’ v3.0. No CR genes were reported earlier in this region, so the candidate gene was identified as a novel gene for clubroot resistance in the present study.

A total of 13 candidate genes were identified in the interval of *Rcr11*, *Rcr13*, and *Rcr_C03-4^ECD10^*. The identified CR QTL from the *B. napus* rutabaga ECD10 will substantially contribute to the development of canola cultivars resistant to emerging strains of *P. brassicae* in the prairie regions of Canada. This study emphasizes the complexity of clubroot resistance in *Brassica napus* and provides valuable insights into the genetic differentiation and specificity of resistance loci. The ability to distinguish between these loci using purified isolates has significant implications for breeding programs targeting durable and isolate-specific resistance.

As whole-genome sequencing of ECD10 is not yet available, the ‘ZS11’ reference genome was used for assembly. A comparison was conducted using eight other *Brassica napus* reference genomes. Although ‘ZS11’ contained the highest number of predicted disease resistance genes across the whole genome (2,708), in the flanking region of *Rcr13*, Shengli had the highest number of resistance genes (38), while ‘ZS11’ contained 22. Similarly, in the *Rcr11* flanking region, Shengli again had the highest count (39), compared to 21 in ‘ZS11’. For the *Rcr_C03-4^ECD10^* flanking region, ‘No2127’ had the highest number of resistance genes (28), whereas ‘ZS11’ contained only one. So some candidate genes may have been missed in the ‘ZS11’ genome. In the current study, the suggested candidate genes are based solely on predicted domains. Future work will include whole-genome sequencing of ECD10, fine mapping of the identified QTL, and functional validation to confirm the roles of candidate genes.

## Data Availability

The raw datasets generated and/or analyzed during this study are available in the National Center for Biotechnology Information (NCBI) repository under BioProject number: PRJNA945869, BioSample number: SAMN33864123, SRA accession numbers: SRR23945225, SRR23945226, SRR23945227 and SRR23945228. https://dataview.ncbi.nlm.nih.gov/?search=SUB12956180&archive=biosample.
